# A structural health monitoring data reconstruction method based on VMD and SSA-optimized GRU model

**DOI:** 10.1038/s41598-025-86781-7

**Published:** 2025-01-28

**Authors:** Xiaoliang Jia, Guoyan Zhang, Zhiqiang Wang, Huacong Li, Jing Hu, Songlin Zhu, Caiwei Liu

**Affiliations:** 1 Shandong Lu Qiao Group CO., LTD, Shandong, 250014 Jinan China; 2 School of Civil Engineering, Qingdao University of Technology, Qingdao, 266525 China; 3 Shandong Zhengyuan Digital City Construction Co., LTD, Shandong, 264670 Shandong Yantai China; 4 Innovation Institute for Sustainable Maritime Architecture Research and Technology, Qingdao University of Technology, Qingdao, 266033 China

**Keywords:** Data Reconstruction, Structural Health Monitoring, Gate Recurrent Unit, Variational Mode Decomposition, Sparrow Search Algorithm, Engineering, Civil engineering

## Abstract

In the field of Structural Health Monitoring (SHM), complete datasets are fundamental for modal identification analysis and risk prediction. However, data loss due to sensor failures, transmission interruptions, or hardware issues is a common problem. To address this challenge, this study develops a method combining Variational Mode Decomposition (VMD) and Sparrow Search Algorithm (SSA)-optimized Gate Recurrent Unit (GRU) for recovering structural response data. The methodology initially employs Variational Mode Decomposition (VMD) to preprocess and decompose the existing data from the target sensor into Intrinsic Mode Functions (IMFs) and residuals. Subsequently, the Gated Recurrent Unit (GRU) network utilizes data from other sensors to reconstruct the IMFs and residuals, ultimately producing the data reconstruction results. During this process, Singular Spectrum Analysis (SSA) is used to optimize the hyperparameters of the GRU network. To validate the effectiveness of this method, we utilized one month of monitoring data collected from a certain project and a publicly available dataset. On the public dataset, we tested performance at different data loss rates. Results show that, compared to a standalone GRU model and a VMD + GRU model, the VMD + SSA + GRU model’s reconstruction data root mean squared error is reduced by 46.61% and 32.57% on average, respectively, while the coefficient of determination increases by 38.74% and 18.50%. The data reconstruction method proposed in this study can accurately capture trends in missing data, without the need for manual hyperparameter tuning, and the reconstruction results are highly consistent with the real data.

## Introduction

With the continuous development of the construction industry in China, large-span spatial structures have been widely applied. However, these structures inevitably suffer from strength degradation and other damages due to factors such as alternating hot and cold conditions, snow and rain loads, wind loads, environmental corrosion, and initial defects during their construction and operation. These issues pose safety hazards. Therefore, ensuring the structural safety and reliability of large-span spatial structures requires long-term monitoring and analysis of structural stress and deformation during construction and operation^1^.

Consequently, structural health monitoring (SHM) has made significant advancements in China. In the SHM field, various sensors are used to monitor structural responses, including but not limited to vibration, displacement, and strain sensors for accurately and effectively assessing the condition of engineering structures. Accurate assessments rely on a large volume of reliable monitoring data. However, due to sensor failures, unexpected damage to data transmission equipment or lines, and unstable power supply during long-term operation of the monitoring system, anomalies and data loss are inevitable. In order to mitigate data loss in structural response monitoring caused by equipment malfunctions, some researchers have expanded upon existing wired sensor networks to develop wireless sensor networks^2^, self-powered wireless sensor networks^3^, and the incorporation of smartphones into structural health monitoring systems^4^. However, these approaches still cannot entirely prevent data loss resulting from sensor or equipment failures. Consequently, a subset of scholars has increasingly focused on and discussed the issue of data restoration within the context of health monitoring engineering. Early commonly used data restoration algorithms include linear regression interpolation, support vector machines (SVM), compressed sensing methods, principal component analysis (PCA), and Bayesian methods. For instance, Ye et al.^5^ proposed a method combining wavelet multiresolution analysis with SVM for reconstructing bridge health monitoring data, showing better effectiveness and accuracy compared to traditional autoregressive moving average (ARMA) methods. Dong et al.^6^ proposed a two-stage SVM-based method to simulate and predict the nonlinear dynamic responses of structures, achieving good accuracy. Jalet et al.^7^ developed a least-squares optimization SVM method for obtaining displacement time series from acceleration data, facilitating displacement data reconstruction when corresponding displacement sensor data is missing. Bao et al.^8^ introduced a new compressed sampling method applied in SHM wireless sensor networks, verifying the accuracy of data loss recovery methods using acceleration time series from the Jinzhou West Bridge and the National Aquatics Center in Beijing, noting decreased recovery accuracy when the original data lacked sparsity in a particular orthogonal basis.

Fereidoun et al.^9^ addressed data loss recovery under varying loss rates through distributed compressed sensing, enhancing reconstruction accuracy and robustness by leveraging correlations between vibration signals. Li et al.^10^ proposed a probabilistic PCA-based data reconstruction method, showing higher accuracy compared to PCA, especially with consecutive missing data. Wan et al.^11,12^ suggested Bayesian modeling using Gaussian processes with a moving window strategy to significantly reduce training data size and computational costs, later proposing a multi-task learning method supported by multi-dimensional Gaussian process priors, achieving good reconstruction performance. Zhang et al.^13^ viewed data recovery as a regression task based on sensor correlations, proposing a Bayesian dynamic regression method with variable regression variables depending on data changes, allowing simultaneous reconstruction of missing data from different sensors. To address the inefficiency of Bayesian time decomposition models with large datasets, Ren et al.^14^ developed an incremental Bayesian/tensor learning scheme, constructing spatiotemporal tensors and performing Bayesian tensor decomposition to extract latent features of missing data, with an incremental learning scheme effectively updating the model, yielding accurate reconstruction results despite extensive random and structured data loss. Zhang et al.^15^ proposed an interpolation method based on point correlations for different data loss scenarios using long-term monitoring data of the steel structure of the Hangzhou Olympic Sports Center Stadium, showing a 5% interpolation error with a correlation coefficient of 0.9 or higher for single-point linear regression, with slightly higher interpolation errors for continuous data loss than for discrete data loss, suggesting the data loss rate should preferably not exceed 30%.

Additionally, deep learning, as proposed by LeCun et al.^16^, has rapidly become one of the most advanced technologies in artificial intelligence in recent years. Compared to traditional machine learning methods, deep learning models consist of multiple processing layers that can learn data representations at various levels of abstraction. These methods have significantly improved technologies in many fields, including speech recognition, visual object recognition, object detection, drug discovery, and genomics. Deep learning uses backpropagation algorithms to uncover complex structures in large datasets and adjust the model’s internal parameters accordingly. Convolutional neural networks (CNNs) excel in handling images, videos, speech, and audio, while recurrent neural networks (RNNs) are more advantageous for processing sequential data such as text and speech. Bao et al.^17^ leveraging the advantages of deep learning in image processing, proposed a data anomaly detection method based on computer vision and deep learning. This method includes two steps: converting data into image vectors through data visualization to create a manually labeled dataset, and then constructing and training a deep neural network for anomaly classification. The results show that this method can automatically detect various patterns of data anomalies with high accuracy. Jeong et al.^18^ recognizing the spatiotemporal correlations among sensor data, proposed a data reconstruction algorithm based on bidirectional recurrent neural networks (BRNNs). This method handles the temporal correlations among sensor data more effectively, demonstrating that the BRNN-based approach outperforms traditional algorithms in accurately reconstructing sensor data. Li et al.^19^ combined empirical mode decomposition (EMD) with long short-term memory (LSTM) neural networks, converting the missing data imputation task into a time series prediction task. EMD helps model the irregular periodic variations in measurement signals, while LSTM can remember long-term associations in subsequences. This approach shows superior performance compared to autoregressive integrated moving average (ARIMA), support vector regression (SVR), and artificial neural network (ANN) models, which are widely used prediction models. Tang et al.^20^ proposed a data recovery algorithm combining Multivariate Variational Mode Decomposition (NVMD) with Fully Convolutional Networks (FCN) and a systematic evaluation model. This algorithm was validated through case studies using measured data, demonstrating its superiority. Subsequently, Tang et al.^21^ further proposed a method to enhance FCNs using residual learning for the recovery of dynamic responses in damaged structures. This method effectively restored the responses of structures under damage conditions, yielding promising results. Sajjad et al.^22^ used a CNN-GRU model to predict short-term residential electricity loads, achieving high predictive accuracy. Hu et al.^23^ proposed a robust precision motion control strategy based on GRU, capturing the dynamic characteristics of motion errors through constructing a GRU neural network. This tracks errors and inputs them into the control system for early compensation, enhancing motion accuracy and delivering satisfactory performance. Wang et al.^24^ proposed a GRU model optimized by the Beluga Whale Optimization (BWO) algorithm to improve laser processing precision, finding that the BWO-GRU model outperformed the BWO-LSTM model. Mateus^25^ compared LSTM and GRU models for predicting the condition of a pulp paper press and found that the GRU model exhibited better performance indicators.

Yang et al.^26^ introduced GRU networks into the field of surgical medicine, using GRU networks to learn the spatiotemporal correlations of points of interest (POIs) and auxiliary points from past data. This method predicts the three-dimensional coordinates of the beating heart’s surface POIs during minimally invasive surgery, thereby enhancing the operability of heart surgery robots. Mim et al.^27^ proposed an initial attention-based GRU network for recognizing human activities, effectively utilizing the temporal and spatial connections in time series data. The proposed model was tested using publicly available datasets and achieved excellent results.

In summary, With the rapid advancement of deep learning technologies, current research on the application of deep learning algorithms in structural health monitoring data reconstruction remains insufficient and presents considerable opportunities for further investigation. GRU networks have found extensive applications across various fields, and some studies have demonstrated that GRUs outperform LSTMs. Therefore, this paper is based on GRU neural networks, fully considering the spatiotemporal correlations between sensors and incorporating Variational Mode Decomposition (VMD) to decompose the irregular periodicities of data signals. This method effectively addresses the stringent limitations of Empirical Mode Decomposition (EMD) regarding data noise and sampling sensitivity, offering strong robustness alongside good performance^28^.During model training, the Sparrow Search Algorithm^29^ (SSA) is introduced to optimize the hyperparameters of the GRU neural network, thereby reducing the time and effort required for manual tuning. Based on the above analysis, this paper adopts the VMD + SSA + GRU optimized approach to achieve strain monitoring data reconstruction by leveraging the spatiotemporal correlations among multiple sensors.

The organization of this paper is as follows: Theoretical background is provided in "Basic methods". "Variational mode decomposition" elaborates on the proposed data reconstruction algorithm. "Sparrow search algorithm" validates the proposed model using a self-built dataset, while "Gate recurrent unit" tests its applicability and reliability with public datasets. Finally, "SHM Data Reconstruction Method Based on VMD + SSA + GRU" and "Results" present the main conclusions and discussions of this paper.

## Basic methods

### Variational mode decomposition

Variational Mode Decomposition (VMD) is a technique for decomposing and estimating signals by minimizing the total bandwidth of all mode estimates. Each mode is assumed to be a signal with a different central frequency and limited bandwidth. The Alternating Direction Method of Multipliers (ADMM) is used to iteratively update the modes and their central frequencies, adjusting each mode to its base frequency band. VMD transforms the signal decomposition process into a non-recursive model, functioning as adaptive Wiener filters and exhibiting strong robustness in noisy environments. It significantly reduces sampling effects compared to Empirical Mode Decomposition (EMD) and Local Mean Decomposition (LMD), and can separate two harmonic signals with close frequencies. In contrast, Singular Value Decomposition (SVD) excels in extracting matrix features with good stability, maintaining minimal impact on singular values despite small changes in matrix elements, and provides scale and rotational invariance to describe mode features^28^.

### Sparrow search algorithm

The Sparrow Search Algorithm (SSA), introduced by Xue^29^ in 2020, is a heuristic optimization algorithm inspired by the foraging and anti-predation behaviors of sparrows. SSA divides the sparrow population into finders, who explore new foraging areas, and joiners, who follow finders. It also considers anti-predation behaviors, where sparrows adjust their positions to evade danger.

Key steps in SSA include initializing the sparrow population, simulating foraging and anti-predation behaviors, evaluating and updating solutions, and determining termination conditions. SSA is known for its simple structure, strong global search capability, and fast convergence, making it suitable for applications in machine learning, intelligent optimization, engineering optimization, and artificial intelligence. It can be used for feature selection, classification, clustering, function optimization, combinatorial optimization, constraint optimization, design optimization, parameter optimization, and control optimization.

### Gate recurrent unit

Gate Recurrent Unit (GRU), proposed by Chung et al.^30^ in 2014, is a variant of the Recurrent Neural Network (RNN) designed to overcome the gradient vanishing problem that traditional RNNs often encounter when processing long sequences of data, and to more effectively capture long-term dependencies within sequences. As an important technology in the field of deep learning, GRU introduces sophisticated gating mechanisms to finely control the flow of information, thereby demonstrating outstanding performance in various application scenarios.

**Fig. 1:**
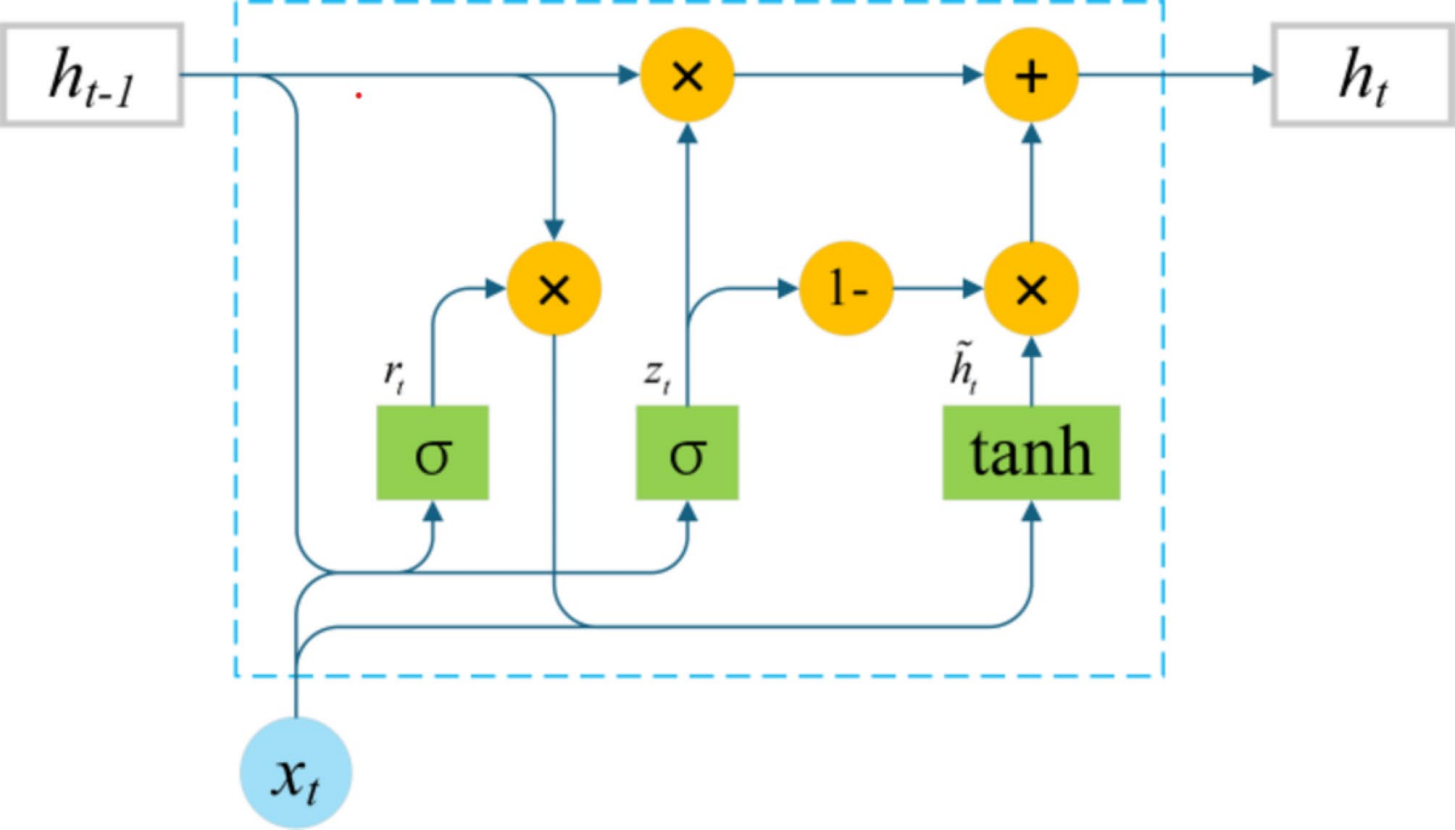
The structure of the GRU.

The core of GRU lies in its two key gating units: the update gate and the reset gate. The structure of the unit is shown in Fig. 1. The update gate determines the extent to which the state information from the previous time step is retained and carried forward to the current state. This mechanism allows the GRU to selectively forget or retain historical information, thus avoiding excessive accumulation or loss of information. The reset gate, on the other hand, controls the degree to which the state information from the previous time step is ignored when computing the current hidden state. Through this mechanism, the GRU can “reset” the hidden state when necessary, better adapting to new input information and thereby enhancing the model’s flexibility and adaptability.

In the network structure of a GRU, the input layer is responsible for receiving external inputs, while the hidden layer processes these inputs and the previous hidden state through gating mechanisms to generate a new hidden state. In this process, the update gate and reset gate play crucial roles. Specifically, the GRU first calculates the values of the reset gate and update gate based on the current input and the previous hidden state. It then uses the reset gate to adjust the influence of the previous hidden state on the candidate hidden state, followed by calculating the candidate hidden state combined with the current input. Finally, the GRU computes the new hidden state based on the update gate, the previous hidden state, and the candidate hidden state, thereby completing the transmission and updating of information.

The formula for calculating GRU units is shown in Eq. (1).

$$\:{r}_{t}=\sigma\:\left({W}_{r}\bullet\:\left[{h}_{t-1},{x}_{t}\right]+{b}_{r}\right)$$

$$\:{z}_{t}=\sigma\:\left({W}_{z}\bullet\:\left[{h}_{t-1},{x}_{t}\right]+{b}_{z}\right)$$

$$\:\stackrel{\sim}{{h}_{t}}=\text{tanh}\left(W\bullet\:\left[{r}_{t}\times\:{h}_{t-1},{x}_{t}\right]+b\right)$$

1 $$\:{h}_{t}=\left(1-{z}_{t}\right)\times\:{h}_{t-1}+{z}_{t}\times\:\stackrel{\sim}{{h}_{t}}$$

Wherein, $\:{r}_{t}$ - Reset gate; $\:{z}_{t}$ - Update gate; $\:W$- Parameter matrix; $\:b$ - Bias vectors; $\:{h}_{t-1}$ - The hidden state of the previous time step; $\:{x}_{t}$ - Current Input; $\:\stackrel{\sim}{{h}_{t}}$ - Candidate hiding status; $\:{h}_{t}$ - Currently hidden.

Compared to other recurrent neural network architectures such as LSTM, GRU offers advantages such as a simpler structure, fewer parameters, and faster training speed. Although LSTM also addresses long-term dependency issues through gating mechanisms, its structure is relatively complex, incorporating multiple gate units including the forget gate, input gate, and output gate. In contrast, GRU achieves similar functionality with just the update gate and reset gate, thereby reducing the complexity and computational cost of the model.

## SHM Data Reconstruction Method Based on VMD + SSA + GRU

**Fig. 2:**
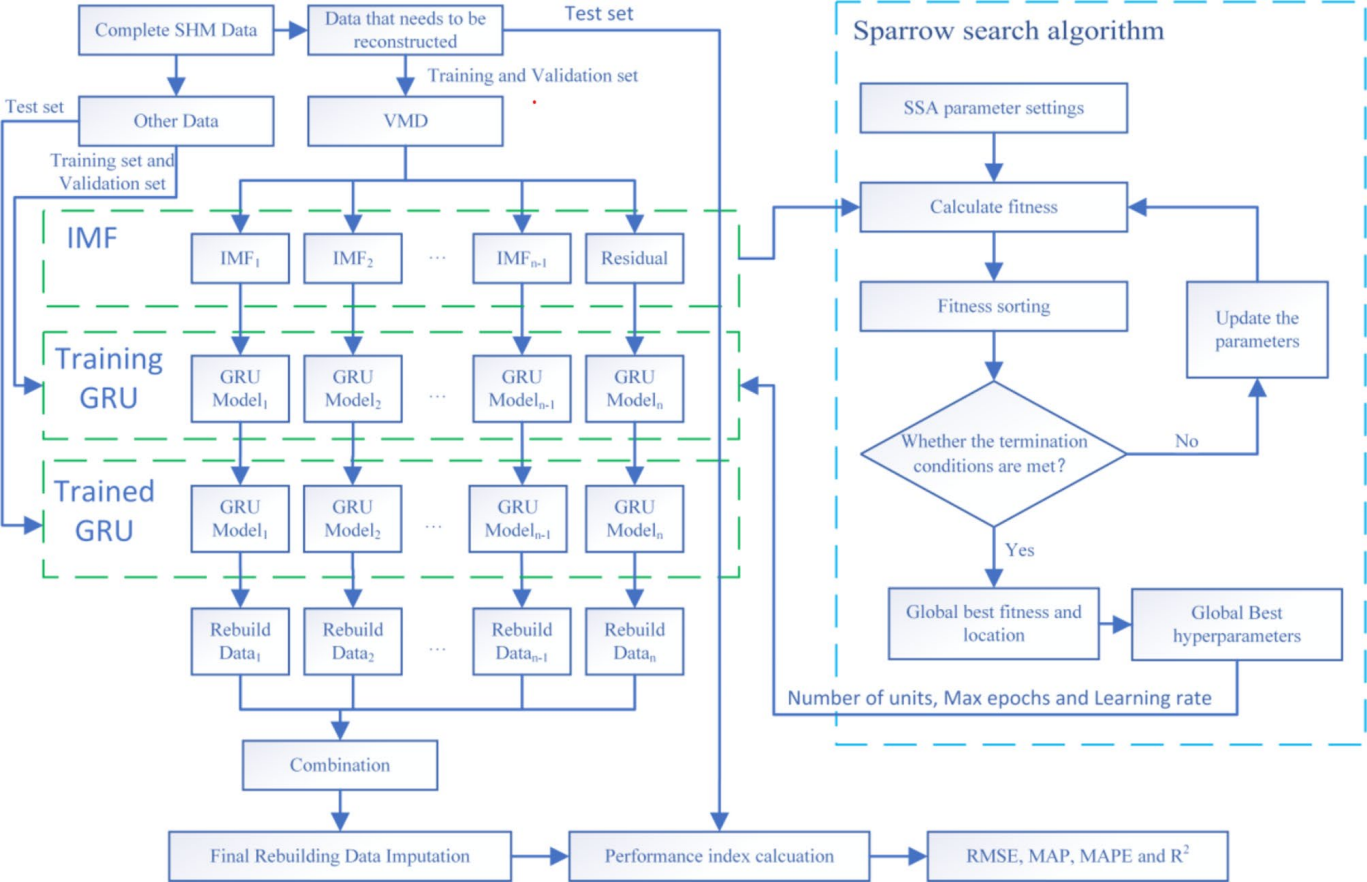
VMD + SSA + GRU model.

Since SHM data involves measuring structural responses at different points within the same building structure, there is inherent correlation among the data. The GRU network possesses a strong advantage in addressing such problems. By preprocessing the data with VMD, the GRU network can better capture the correlations within the data. Additionally, incorporating the SSA to optimize the training parameters of the GRU model can enhance the data reconstruction performance without manual intervention. Consequently, this paper employs a VMD and SSA-optimized GRU model to reconstruct the missing parts of the SHM data. The methodological framework of this approach is illustrated in Fig. 2.

Step 1: First, determine the target sensor data loss rate and the proportion of the validation set relative to the entire dataset, assumed here to be 20% and 10%, respectively. Perform VMD on the first 70% of the target sensor data. Normalize each Intrinsic Mode Function (IMF) component, the residual, and all complete data from other sensors obtained after decomposition.

Step 2: Construct the training set, validation set, and test set. Use the first 70% of other sensors’ data as the training set input, the middle 10% as the validation set input, and the last 20% as the test set input. The corresponding 70% of the target sensor’s IMF components and residual are used as the training set output, and the corresponding middle 10% as the validation set output. Establish a GRU model for each IMF component and residual of the target sensor.

Step 3: Initialize the relevant parameters of the SSA. Input part of the data into the model for parameter training, determining the three hyperparameters: number of hidden layer units, number of iterations, and learning rate, to achieve the best structure for data fitting and prediction.

Step 4: Train the GRU models for each IMF component and residual of the target sensor, denormalize and sum the obtained results to get the final prediction result. Perform error analysis by comparing this result with the original data of the target sensor to evaluate the model’s data reconstruction performance.

In this paper, the Root Mean Square Error (RMSE), Mean Absolute Error (MAE), Mean Absolute Percentage Error (MAPE), and coefficient of determination (R^2^) are used to evaluate the prediction performance. These metrics are defined as follows:

2 $$\:RMSE=\sqrt{\frac{1}{n}\sum\:_{i=1}^{n}{\left({t}_{i}-{y}_{i}\right)}^{2}}$$

3 $$\:MAE=\frac{1}{n}\sum\:_{i=1}^{n}\left|{t}_{i}-{y}_{i}\right|$$

4 $$\:MAPE=\frac{100\%}{n}\sum\:_{i=1}^{n}\left|\frac{{t}_{i}-{y}_{i}}{{y}_{i}}\right|$$

5 $$\:{R}^{2}=1-\frac{\sum\:_{i=1}^{n}{\left({y}_{i}-{t}_{i}\right)}^{2}}{\sum\:_{i=1}^{n}{\left({t}_{i}-\stackrel{-}{t}\right)}^{2}}$$

Where $\:{t}_{i}$ is the measured value, $\:{y}_{i}$ is the predicted value, $\:\stackrel{-}{t}$ is the average value of the measured value.

## Results

In this section, we take the steel structure roof of a project as an example to verify the accuracy and effectiveness of the combined model based on VMD, SSA and GRU for the reconstruction of the missing part of the SHM data.

### Project description

The main subject of this study is a public transportation building (4E class airport, class one terminal) with a total construction area of 121,680 square meters. The building comprises three floors above ground, with partial sections having four and five floors, and one partial underground level. The steel structure is mainly distributed in the hall, atrium skylight roof, canopy, and mezzanine within the building. The component types include steel columns, steel beams, fork columns, cast steel joints, roof plane trusses, triangular tube trusses, and other components.

The terminal building’s steel roof has planar dimensions of 342.6 m by 145.3 m, utilizing a combination of plane trusses and triangular tube trusses. The roof has an undulating wave-like shape, with the highest installation elevation at + 35.5 m. The roof structure is supported by steel tube concrete columns, including Y-shaped fork columns, rocking columns, and vertical columns.

**Fig. 3:**
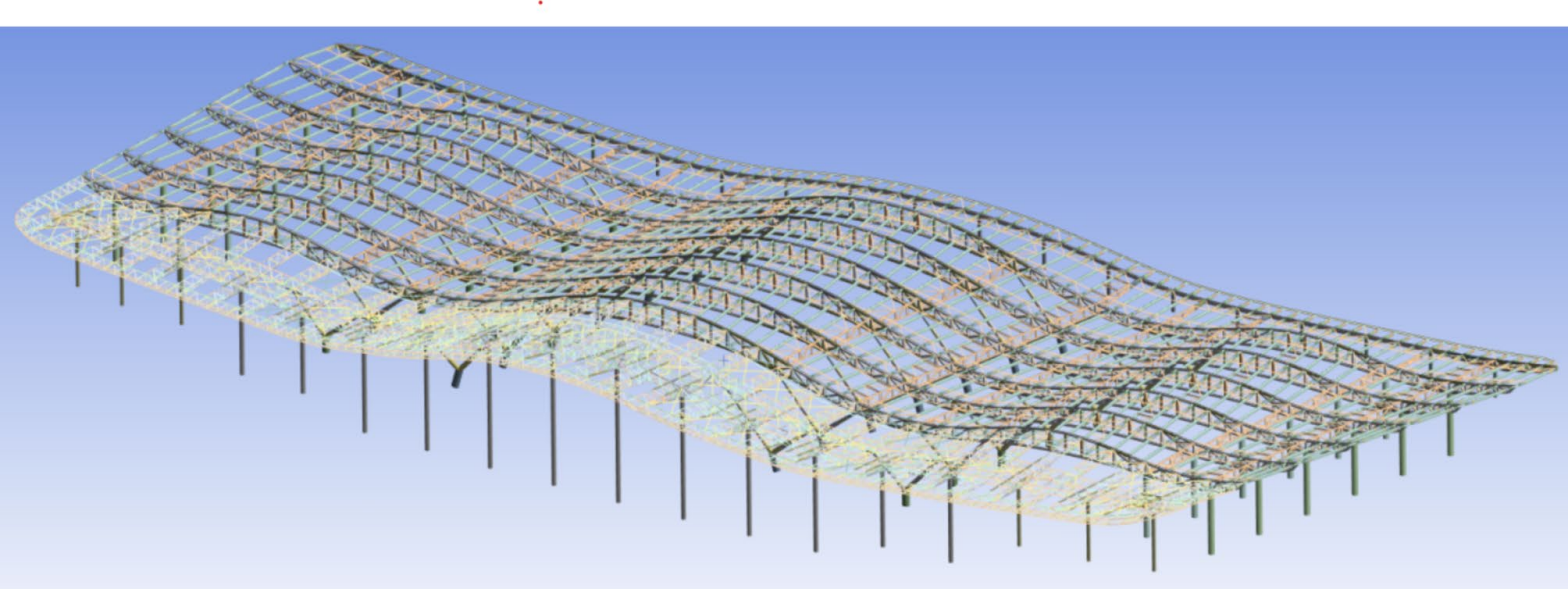
Model of the Steel Roof Structure of the Terminal Hall.

**Fig. 4:**
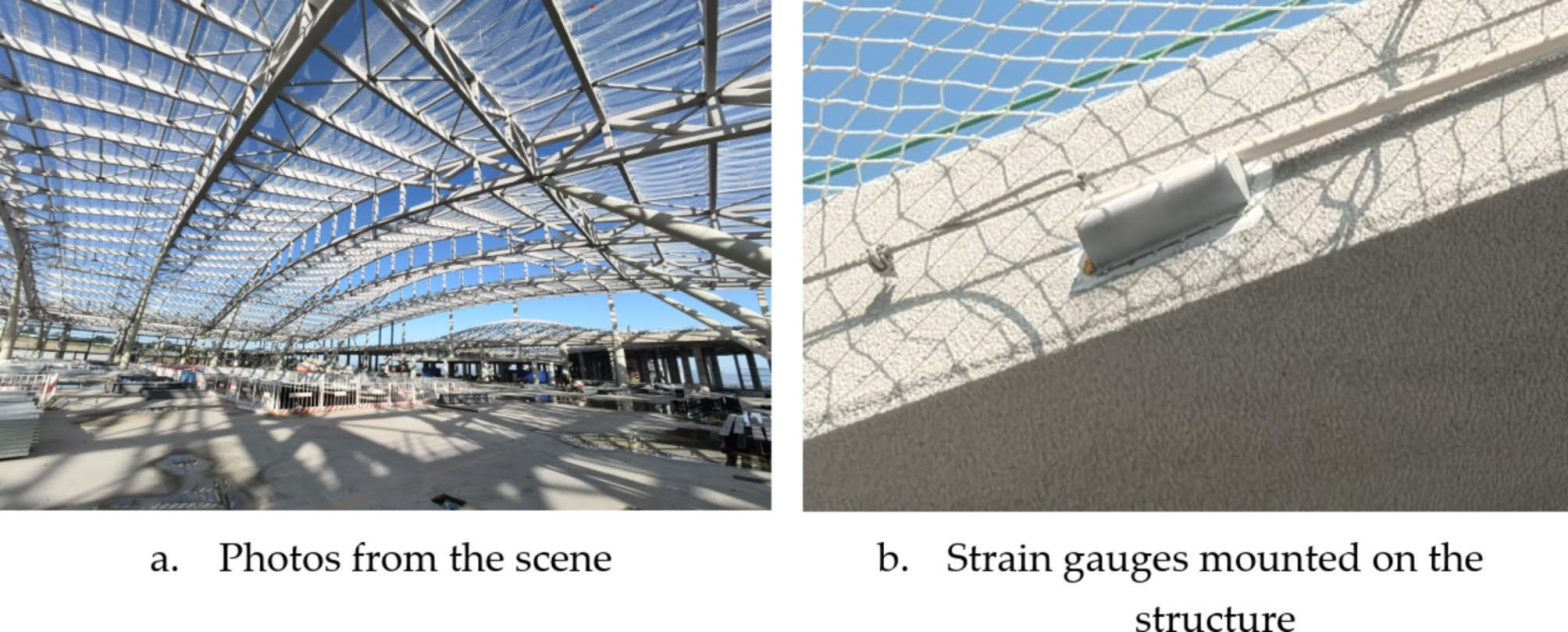
Stress-strain sensors mounted on steel roofing elements.

The hall’s planar trusses mainly consist of steel trusses, box-type tie beams, and steel tie rods. The main planar trusses have a maximum span of 63 m and a maximum overall length of 342 m, with a total of 6 trusses. The main trusses feature an arc-shaped structure with heights ranging from 2100 mm to 5000 mm. The secondary planar trusses are 16.2 m in length, with a maximum single-span weight of 6 tons, totaling 72 trusses. These trusses connect the main trusses and are positioned along the column support axis, all made of box-type components. There are 37 triangular tube trusses, with the inner side rigidly connected to the planar trusses and the front end connected to the rocking column pin shaft. The maximum cantilever length is 18 m, and the maximum single truss length is 45 m. The structural model of the terminal hall is shown in Fig. 3, and the on-site installed vibrating wire strain gauges are depicted in Fig. 4. The specific parameters of the vibrating wire strain gauges used in this project are detailed in Table 1.

**Table 1:** Vibrating wire strain gauge parameters.

Performance	Parameter
Measuring range	3000$\:\mu\:\epsilon\:$
Accuracy	1%FS
Protection level	IP68
Non-linearity	Straight line: ≤1%FS;
	Polynomial: ≤0.1%FS
Resolution	0.035%FS
Temperature range	−20 °C to + 80 °C
Gauge length	150 mm

### Data sample

**Fig. 5:**
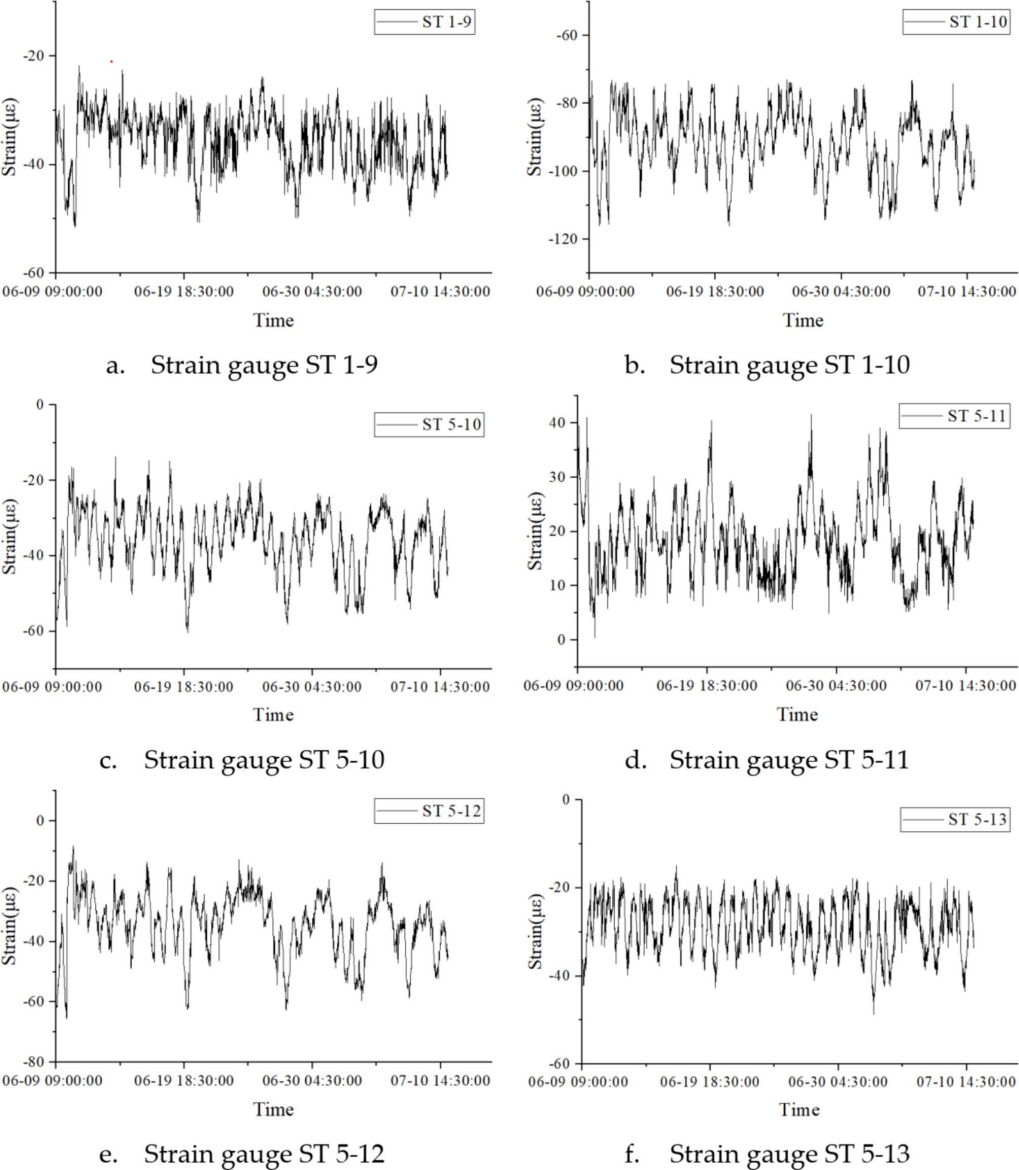
Raw data strain–time history diagrams.

The data sample for this study consists of the complete dataset from six out of the 166 strain sensors installed on the steel roof of the terminal hall between June 9, 2023, and July 11, 2023. The data type is strain response. The missing data systematically simulates the loss of data from individual sensors due to unexpected damage or failure. Figure 5 displays the strain-time curves for these six vibrating wire strain gauges.

Assume that the last 10–70% (at intervals of 5%) of the data from sensor ST 5–13 is missing. Construct the corresponding training set, validation set, and test set. Use the pure GRU neural network, the VMD + GRU neural network, and the VMD + SSA + GRU neural network to repair the missing data. Evaluate the reconstruction results for each of the aforementioned datasets.

### Model settings

**Fig. 6:**
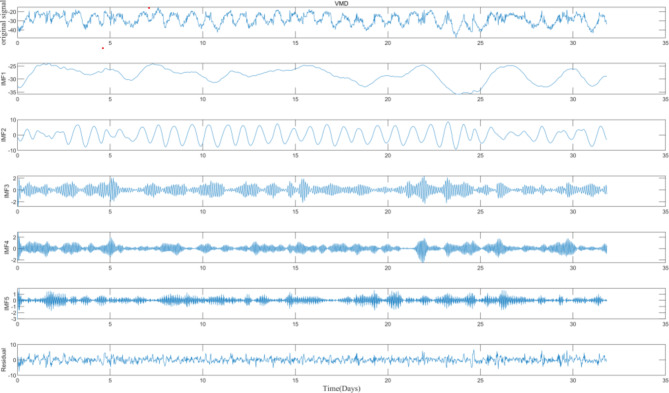
ST5-13 whole raw data and IMF components and residuals.

Perform VMD on the information from the target sensor (ST 5–13), decomposing it into five Intrinsic Mode Function (IMF) components and one residual component, as illustrated in Fig. 6.

The initial parameter settings for the GRU model are as follows: the GRU has one layer, and training is conducted using the Adam optimizer. The maximum number of training epochs is 30, with an initial learning rate set at 0.01. The learning rate is adjusted once every 6 training iterations, with an adjustment factor of 0.2. The regularization parameter is 0.01. The model comprises 70 hidden units and utilizes the ReLU function as the activation function. A subset of the data is used for pre-training, after which inference evaluation is performed. The SSA is used to optimize three hyperparameters—number of units, training iterations, and learning rate—based on the results and the corresponding model parameters.

### Reconstruction results

Table 2 shows the hyperparameters of the GRU model optimized using SSA at different missing data rates. From the data in the table, it can be observed that as the missing rate increases, there is a certain upward trend in the maximum number of training epochs, while the number of hidden layer units and the initial learning rate exhibit significant fluctuations.

**Table 2:** Hyperparameters of the GRU model optimized using SSA for target sensor (ST5-13).

Missing data rate	Number of units	Max epochs	Learning rate
10	440	110	0.00528
15	465	73	1E-4
20	872	226	1E-4
25	945	103	0.0014
30	50	116	0.0026
35	471	107	0.00281
40	946	60	1.72216E-4
45	371	191	0.00171
50	248	260	0.00587
55	444	257	0.00554
60	345	249	0.00599
65	66	291	0.00876
70	238	203	0.0023

**Fig. 7:**
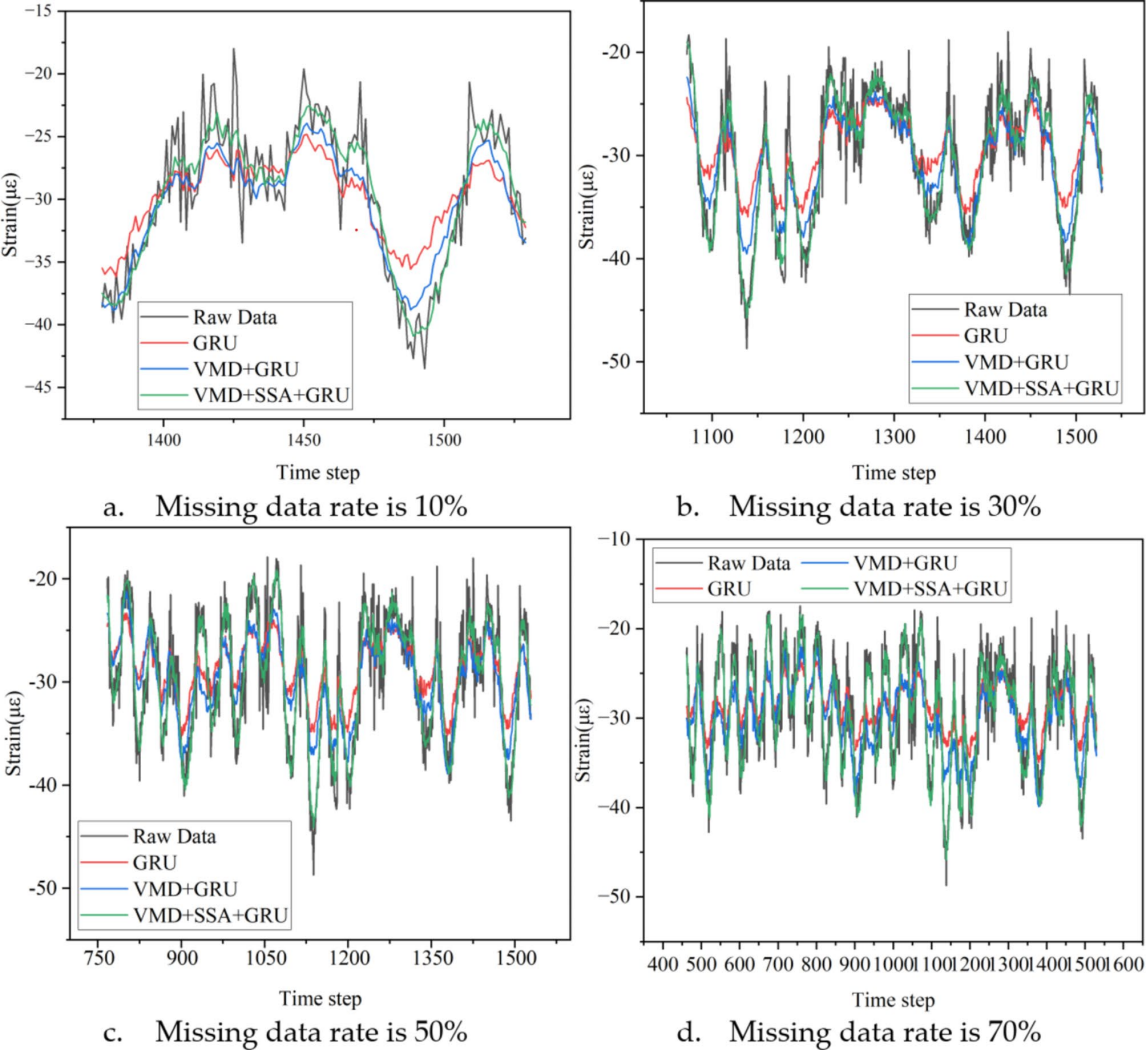
Partial reconstruction results of target sensor (ST5-13) under different missing rates.

Figure 7 shows a comparison of the effectiveness of three algorithms—namely GRU, VMD + GRU, and VMD + SSA + GRU—in reconstructing missing sensor data using normal sensor data across 13 different missing data rate scenarios, ranging from 10 to 70% in 5% increments. This figure provides a partial display of the comparison results.

Figure 7 shows that the reconstructed data obtained through the three methods is consistent with the original data in overall trends. However, some errors can be observed in peak areas where the signal changes sharply. Notably, when using the GRU method alone for data reconstruction, a significant smoothing effect occurs, where the reconstructed data tends to approximate the local average value near the local maxima and minima. In contrast, the data reconstructed using the VMD + SSA + GRU method is closer to the actual data, with relatively smaller errors.

**Fig. 8:**
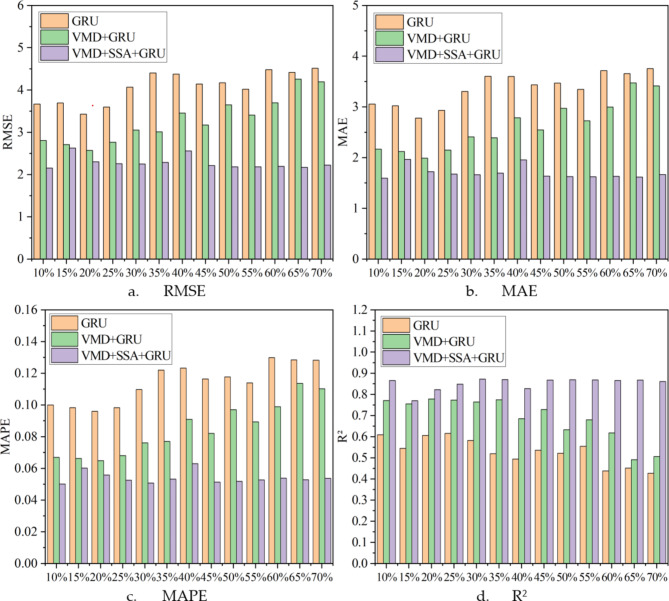
The evaluation results of the reconstructed data of the target sensor (ST5-13) by three different data reconstruction algorithms under different evaluation indicators.

**Fig. 9:**
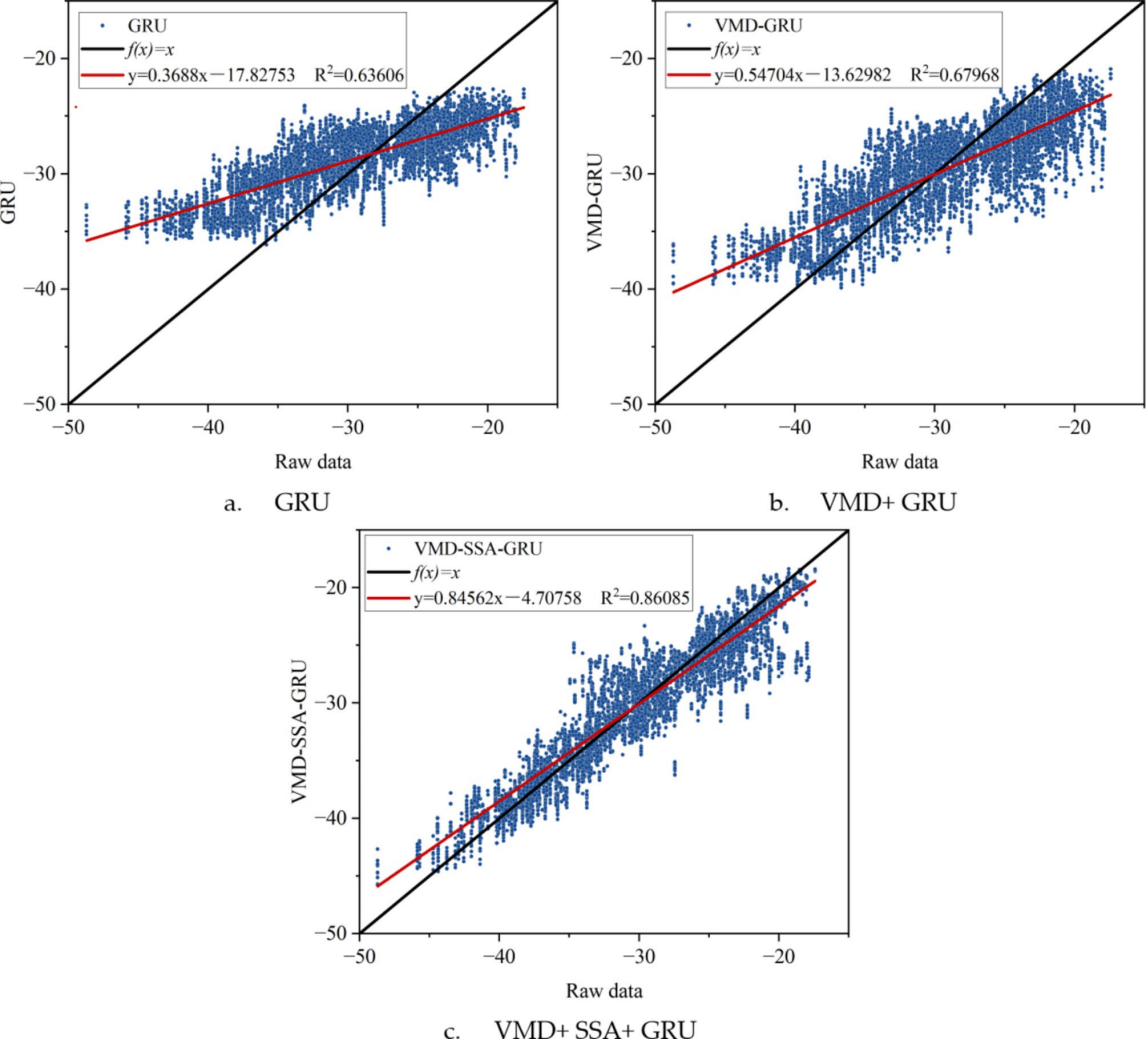
Scatter plots and linear regression graphs of data reconstruction results of target sensor (S5-13) using three different algorithms.

**Fig. 10:**
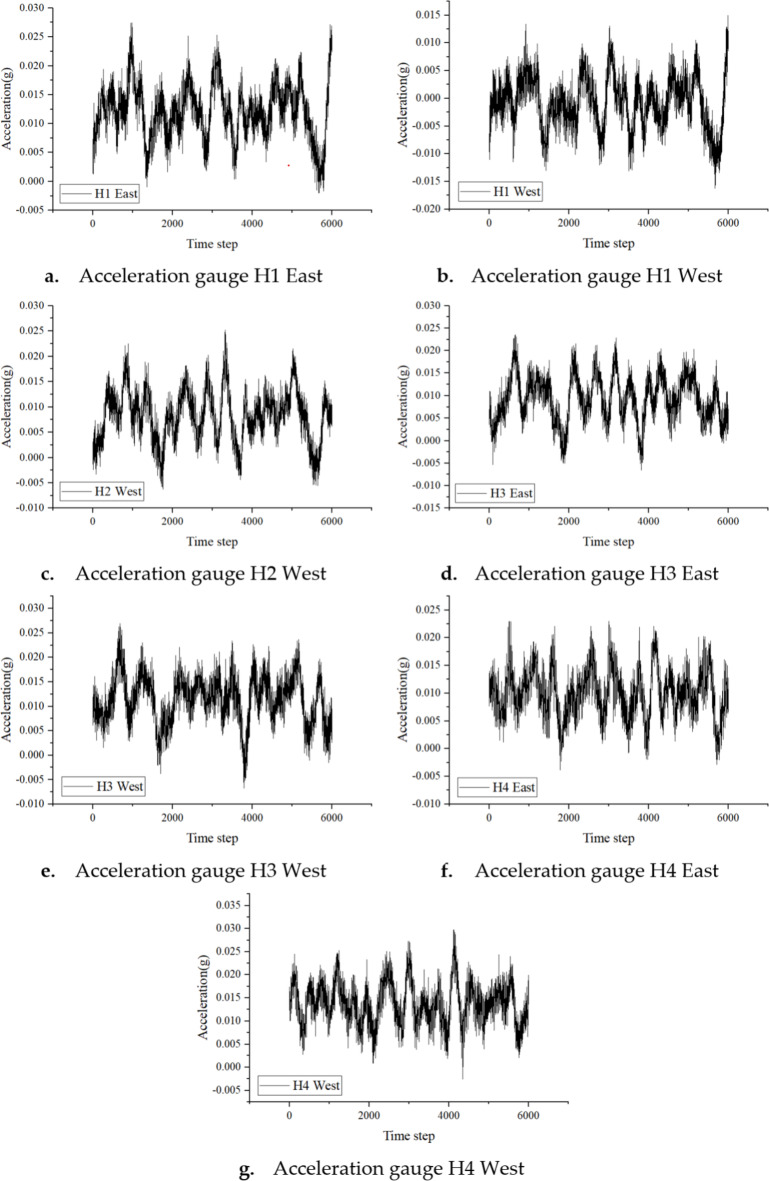
Raw data time history diagram.

To more clearly compare the differences between the reconstructed data and the original data under different conditions using various algorithms, multiple evaluation metrics were used to assess the performance of each algorithm, and the evaluation results are presented in Fig. 8 for intuitive lateral comparison (Fig. 9). Additionally, to explore whether the reconstruction error is related to its magnitude, Fig. 10 was specially drawn to visualize the error between the reconstructed data and the original data under all conditions.

A lateral comparison of the evaluation results, as shown in Fig. 8, indicates that an increase in the data loss rate leads to a proportional rise in the loss rate. Figure 9 displays the overall effectiveness of data reconstruction using three different methods under varying degrees of data loss. Notably, the VMD + SSA + GRU model exhibits the lowest sensitivity to increasing loss rates, highlighting the effectiveness of using VMD for decomposing the initial monitoring data. Additionally, optimizing the hyperparameters of the GRU model with SSA significantly improves the accuracy of data reconstruction.

From Fig. 9, it can be clearly seen that the GRU network exhibits a significant tendency towards the dataset’s mean value when reconstructing stress data. Specifically, when the original data approaches the maximum value, the reconstructed data tends to decrease, and when the original data approaches the minimum value, the reconstructed data tends to increase. This central convergence trend is considered the primary cause of errors.

Notably, VMD effectively decomposes the data into multiple intrinsic mode functions (IMFs), aiding the GRU network in better understanding the spatiotemporal relationships within the data. Therefore, the application of VMD helps mitigate the central convergence tendency of extreme data points. Furthermore, optimizing the GRU network with SSA further alleviates this convergence trend, achieving more precise reconstruction results.

Under 13 different data loss conditions, the VMD + SSA + GRU method demonstrates significant improvements in data reconstruction performance. Compared to a standalone GRU method and the VMD + GRU combination method, this approach reduces the RMSE (Root Mean Square Error) by an average of 44.11% and 30.74%, respectively. Additionally, the MAE (Mean Absolute Error) is reduced by an average of 49.48% and 35.37%, while the MAPE (Mean Absolute Percentage Error) is reduced by 52.64% and 36.27%. Furthermore, the R^2^ (coefficient of determination) increases by an average of 37.70% and 19.13%, respectively, further proving the effectiveness of the VMD + SSA + GRU method.

## Public dataset validation

### Dataset source

The publicly available dataset used in this section comes from the long-term monitoring project of the Hardanger Bridge conducted by the Norwegian University of Science and Technology. This dataset is openly accessible^31^.

### Data samples

The data used in this study originates from seven accelerometers deployed along the z-axis of the Hardanger Bridge during the Ole storm on February 7, 2015. The specific sensors include H1 East, H1 West, H2 West, H3 East, H3 West, H4 East, and H4 West. The original data from these sensors is displayed in Fig. 10, with time steps on the horizontal axis and acceleration values on the vertical axis. The data was sampled at a frequency of 200 Hz, with a continuous collection duration of 30 s.

Assume that the last 10–70% (at intervals of 5%) of the data from the H4 West accelerometer is missing. Construct the corresponding training set, validation set, and test set. Use the pure GRU neural network, the VMD + GRU neural network, and the VMD + SSA + GRU neural network to repair the missing data. Evaluate the reconstruction results for each of the aforementioned datasets.

### Model settings

**Fig. 11:**
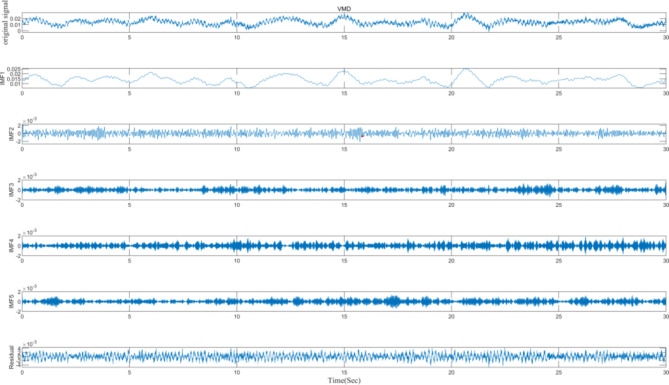
H4 West whole raw data and IMF components and residuals.

Perform VMD on the information from the target sensor (H4 West) under different conditions of loss rates, decomposing it into five Intrinsic Mode Function (IMF) components and one residual component. The VMD results without any missing data are shown in Fig. 11.

The initial settings for the GRU model are as follows: the hidden layer consists of one GRU layer, trained using the Adam optimization algorithm. The maximum number of training iterations is set to 100, with an initial learning rate set at 0.01. The learning rate is updated every 20 training iterations using a learning rate adjustment factor of 0.2. Additionally, the regularization parameter is set to 0.01. The model includes 70 hidden units and employs the ReLU function as the activation function. During training, we first pre-train the model using a subset of the dataset, and then evaluate it upon completion of training. To further optimize the model, we use the SSA to adjust and optimize three hyperparameters: the number of hidden units, the number of training iterations, and the learning rate, based on the pre-training evaluation results and model parameters.

### Reconstruction results

Table 3 shows the parameters of the GRU model optimized using SSA under different data loss conditions. From the data in the table, it can be observed that as the data loss rate increases, the number of units in the SSA-optimized GRU model fluctuates significantly, while the maximum number of training iterations and the initial learning rate exhibit an upward trend.

**Table 3:** Hyperparameters of the GRU model optimized using SSA for target sensor (H4 west).

Missing data rate	Number of units	Max epochs	Learning rate
10	319	246	1E-4
15	704	21	6.06401E-4
20	280	74	1.12125E-4
25	229	129	0.00322
30	727	209	0.00162
35	389	250	0.00327
40	355	201	0.00272
45	497	237	0.00181
50	646	166	0.00407
55	628	187	0.00475
60	131	79	0.00173
65	266	25	8.37818E-4
70	384	262	6.76796E-4

**Fig. 12:**
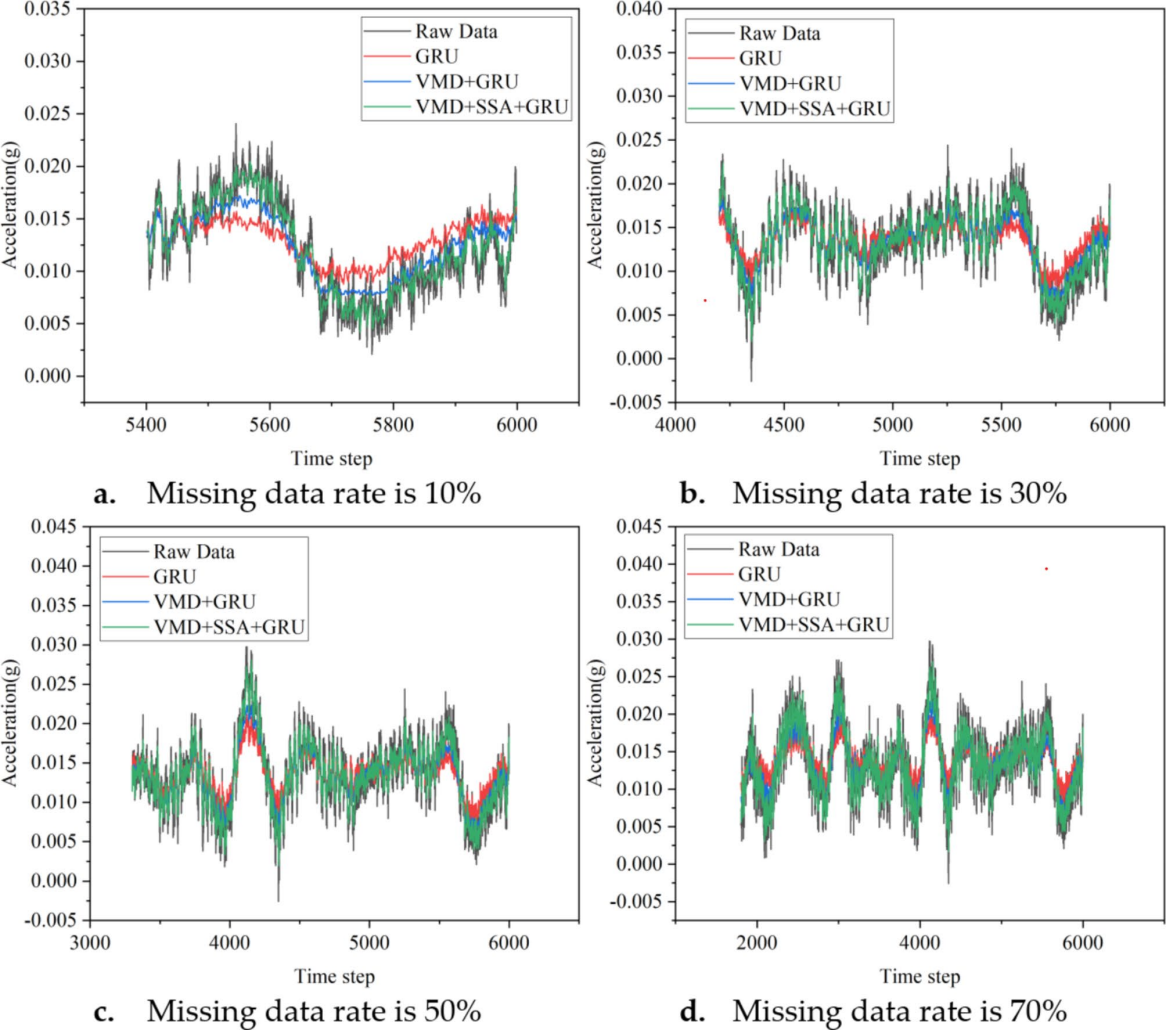
Partial reconstruction results of target sensor (H4 West) under different missing rates.

Figure 12 illustrates the comparison of the effectiveness of three algorithms—namely GRU, VMD + GRU, and VMD + SSA + GRU—in reconstructing missing sensor data using normal sensor data across 13 different data loss rate scenarios, ranging from 10 to 70% in 5% increments. This figure provides a partial display of the comparison results.

Figure 12 shows that the reconstructed data obtained through three different methods largely align with the original data trends. However, some deviations can be observed in areas where the signal peaks change sharply. Notably, when using the GRU method alone for reconstruction, there is a significant smoothing effect, where the reconstructed data tends to approximate the local mean near the local maxima and minima. In contrast, the reconstructed data obtained using the combined VMD, SSA, and GRU method fits the actual data more closely, with relatively smaller errors.

**Fig. 13:**
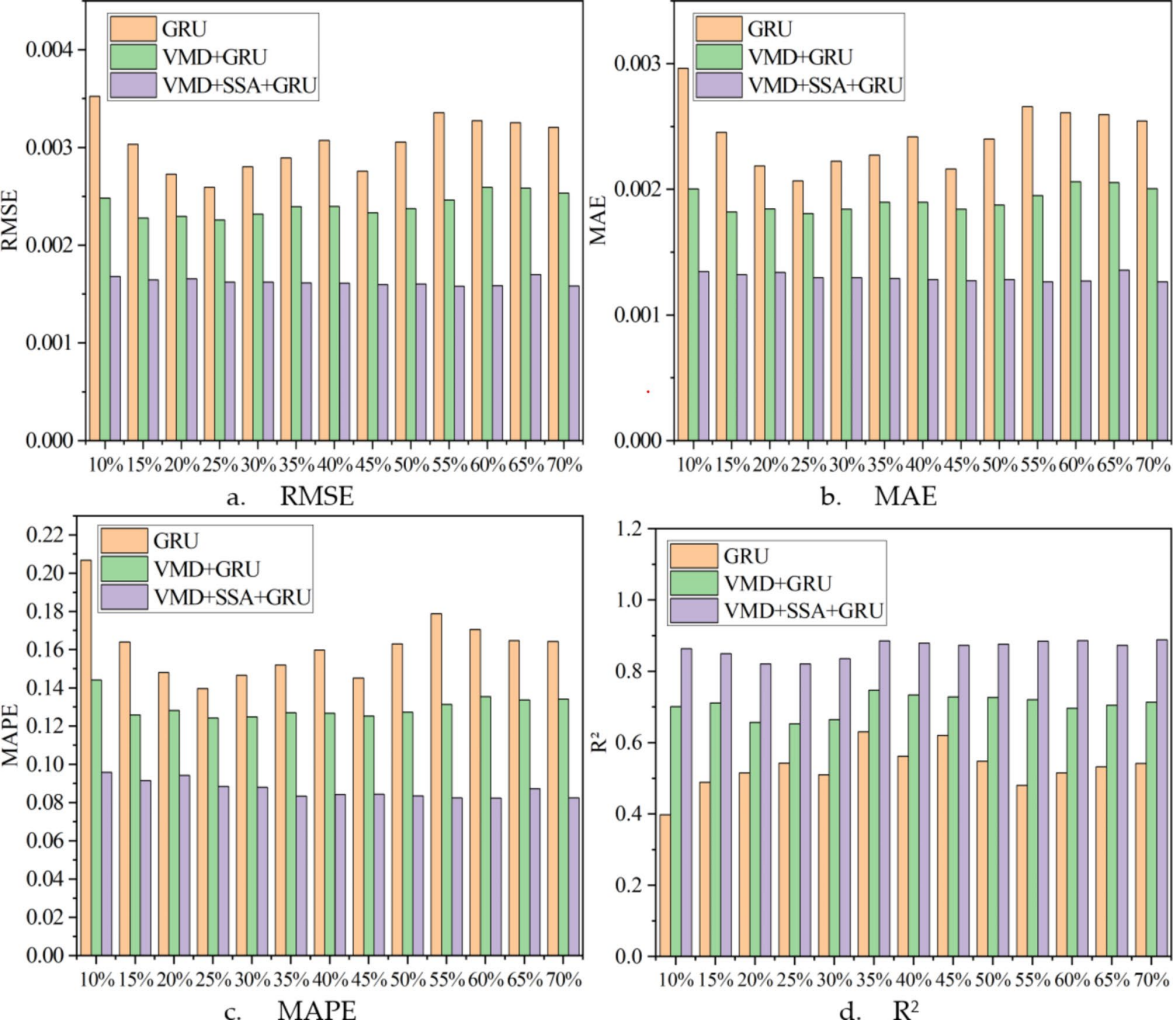
The evaluation results of the reconstructed data of the target sensor (H4 West) by three different data reconstruction algorithms under different evaluation indicators.

**Fig. 14:**
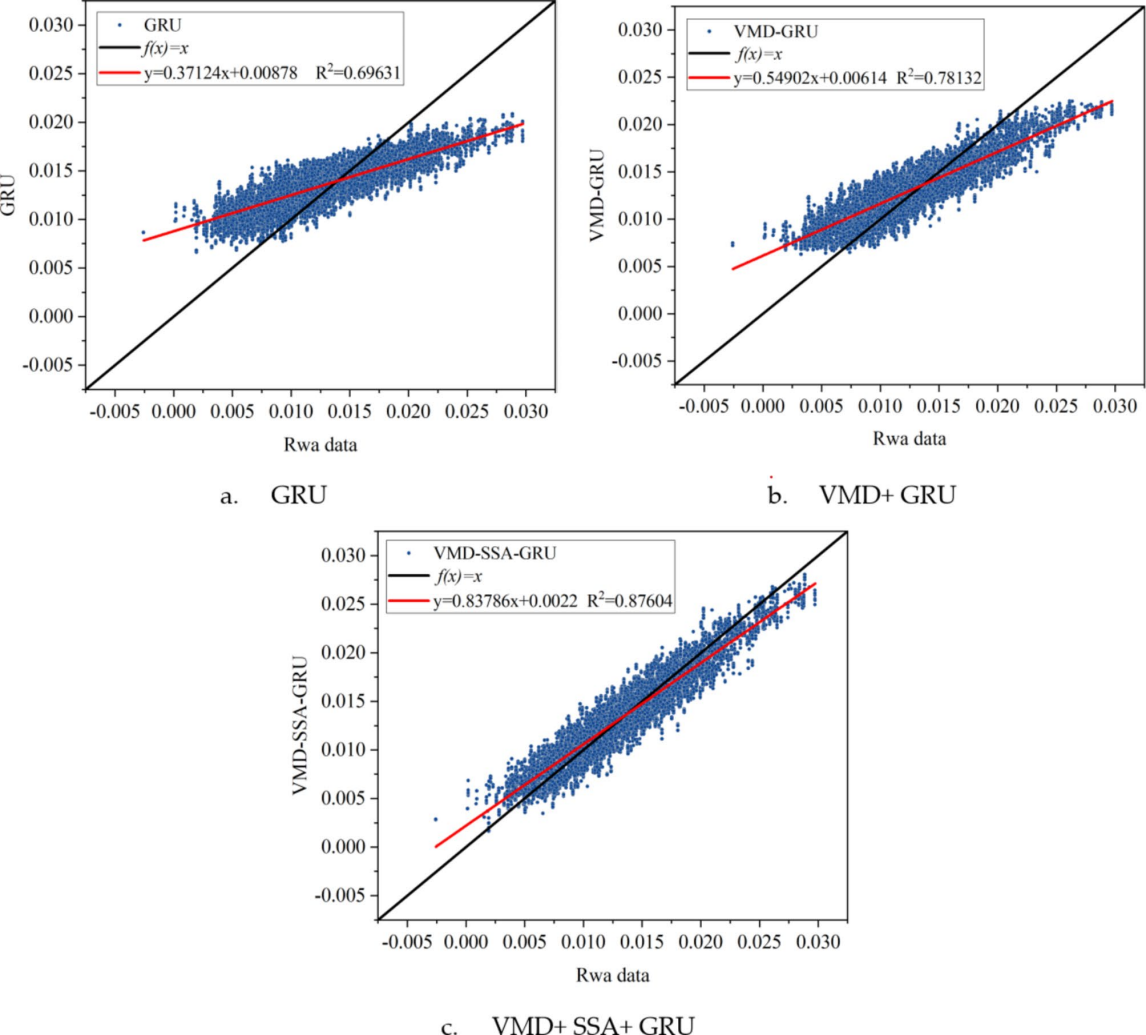
Scatter plots and linear regression graphs of data reconstruction results of target sensor (H4 West) using three different algorithms.

To more clearly show the differences between the reconstructed data and the original data under different evaluation criteria, the output results of various algorithms under different conditions were evaluated using multiple metrics. These results are comprehensively displayed in Fig. 13 for intuitive lateral comparison. Additionally, to explore whether the reconstruction error is related to its magnitude, Fig. 14 was specially created to visualize the error between the reconstructed data and the original data under all conditions.

Figure 13 illustrates the lateral comparison of the evaluation results. It is evident from the figure that as the data loss proportion increases, the error in reconstructed data also shows a corresponding upward trend. This figure compares the performance of three different data reconstruction methods under various data loss rates. Notably, the VMD + SSA + GRU combined model exhibits the lowest sensitivity to increasing data loss rates, strongly demonstrating the significant effectiveness of VMD in the preliminary processing of monitoring data. Additionally, by finely tuning the hyperparameters of the GRU model using SSA, we found that the accuracy of the GRU model in data reconstruction has been further improved.

Figure 14 clearly shows a significant trend in the GRU network’s reconstruction of acceleration data: the data tends to converge towards the mean value of the entire dataset. Specifically, when the original data is near its maximum value, the GRU-reconstructed data tends to decrease; conversely, when the original data is near its minimum value, the reconstructed data tends to increase. This central convergence phenomenon is considered a major cause of errors.It is worth noting that the application of VMD effectively decomposes the data into multiple intrinsic mode functions, aiding the GRU network in better understanding the spatiotemporal correlations within the data. This step helps mitigate the trend of extreme data points converging towards the mean. Furthermore, optimizing the GRU network with SSA alleviates this convergence trend to a greater extent, resulting in more accurate reconstruction outcomes.

Under 13 different data loss conditions, the VMD + SSA + GRU method demonstrates significant improvements in data reconstruction performance. Compared to a standalone GRU method and the VMD + GRU combination method, this approach reduces RMSE (Root Mean Square Error) by an average of 46.61% and 32.57%, respectively. Additionally, the MAE (Mean Absolute Error) is reduced by an average of 46.47% and 32.14%, while the MAPE (Mean Absolute Percentage Error) is reduced by 46.35% and 33.16%. Furthermore, the R^2^ (coefficient of determination) increases by an average of 38.74% and 18.50%, respectively, further proving the effectiveness of the VMD + SSA + GRU method.

## Conclusions

In the field of SHM, ensuring the completeness and accuracy of data is crucial for reliably assessing the safety of building structures. Addressing the challenge of frequent strain sensor data loss in long-span spatial SHM systems, this study proposes an innovative method that reconstructs the missing strain sensor data using data from other sensors within the structure. The core of this method lies in combining VMD technology with SSA optimization to finely tune the hyperparameters of the GRU neural network. To validate the effectiveness of this method, strain monitoring data from an actual engineering project and acceleration sensor data from the Hardanger Bridge in Norway were used. The main conclusions are as follows:

1. This method successfully addresses the difficulty of manually tuning hyperparameters in a single deep learning algorithm model, significantly saving the time and effort required for optimizing model hyperparameters. However, it is worth noting that the SSA used in this study is only one of many hyperparameter optimization methods and may risk getting trapped in local optima rather than global optima, which could impact the model’s effectiveness.
2. Verification using SHM data from an actual engineering project confirmed that the proposed method accurately captures and reconstructs the trends in missing data, with reconstruction results highly consistent with the real data. Additionally, this method also showed excellent reconstruction performance and generalization ability when handling acceleration response data from long-span suspension bridges.
3. Compared to the standalone GRU method and the combination of VMD and GRU, the VMD + SSA + GRU method achieved significant success in reconstructing strain data. Specifically, it reduced the RMSE (Root Mean Square Error) by an average of 44.11% and 30.74%, reduced MAE (Mean Absolute Error) by 49.48% and 35.37%, and reduced MAPE (Mean Absolute Percentage Error) by 52.64% and 36.27%. Meanwhile, the R² (coefficient of determination) saw an average increase of 37.70% and 19.13%. In terms of reconstructing acceleration data, the VMD + SSA + GRU method also demonstrated clear advantages. Compared to GRU and VMD + GRU, this method reduced the RMSE by an average of 46.61% and 32.57%, reduced MAE by 46.47% and 32.14%, and reduced MAPE by 46.35% and 33.16%. Notably, the R² increased by an average of 38.74% and 18.50%. In summary, the VMD + SSA + GRU data reconstruction method proposed in this study not only provides higher accuracy and lower error rates but also significantly reduces sensitivity to data loss rates. Given sufficient training data, this method exhibits outstanding stability during the data reconstruction process.

## Discussion

Although the VMD and SSA-optimized GRU model proposed in this paper has achieved significant improvements in data reconstruction accuracy, there remains a risk of GRU hyperparameters falling into local optima, which can affect the attainment of better global reconstruction outcomes. In the future, more intelligent optimization algorithms that further mitigate the risk of local optima could be introduced to obtain a more reliable data reconstruction model.

The method proposed in this paper has only been validated on the stress-strain data of large-span steel structures and the acceleration data of suspension bridges, without further validation on other structural responses to prove its applicability. Additionally, the basic units of GRU have not been optimized. With the enhancement of computational capabilities in the future, more computationally intensive neural network algorithms could be employed for data reconstruction. As the number of model parameters increases, the ability to capture intrinsic data patterns will be strengthened, leading to improved reconstruction accuracy.

## Data Availability

All data generated or analyzed during this study are included in this published article.
